# Cellular and Molecular Characteristics of Scarless versus Fibrotic Wound Healing

**DOI:** 10.1155/2010/790234

**Published:** 2010-12-27

**Authors:** Latha Satish, Sandeep Kathju

**Affiliations:** ^1^Center for Genomic Sciences, Allegheny-Singer Research Institute, Allegheny General Hospital, 320 East North Avenue, Pittsburgh, PA 15212, USA; ^2^Department of Microbiology and Immunology, Drexel University College of Medicine, Allegheny Campus, Pittsburgh, PA 15212, USA

## Abstract

The purpose of this paper is to compare and contrast the discrete biology differentiating fetal wound repair from its adult counterpart. Integumentary wound healing in mammalian fetuses is essentially different from wound healing in adult skin. Adult (postnatal) skin wound healing is a complex and well-orchestrated process spurred by attendant inflammation that leads to wound closure with scar formation. In contrast, fetal wound repair occurs with minimal inflammation, faster re-epithelialization, and without the accumulation of scar. Although research into scarless healing began decades ago, the critical molecular mechanisms driving the process of regenerative fetal healing remain uncertain. Understanding the molecular and cellular events during regenerative healing may provide clues that one day enable us to modulate adult wound healing and consequently reduce scarring.

## 1. Introduction

In adult (postnatal) mammalian organisms, injury to cutaneous tissue with disruption of normal skin architecture is repaired by means of an inflammatory and fibrotic response that leads to accumulation of scar [1]. Although scar formation allows for the rapid sealing of an injured area, it can frequently prove the source of persistent pathology in the organism. For example, scar formation after tendon repair will limit their gliding ability, restricting hand function; intra-abdominal scar/adhesions frequently lead to small bowel obstruction, necessitating surgical intervention; cirrhosis of the liver and pulmonary fibrosis are also forms of excessive scarring. 

Nowhere, however, is scar more evident or problematic than in the skin. Cicatrix in the extremities and digits can cause contracture and restrict motion, resulting in significant disability. Scar in the genitalia can interfere with sexual function and even urination. Scar formation in the facial skin of the head and neck is particularly problematic, with multiple vital functions at risk. Scar in the external ear can cause substantial hearing loss, and constriction of the nasal apertures can interfere with respiration, smell, and derivatively, taste. Scar contractures following burn injury are well known to progress to microstomia, nasal stenosis, lip or eyelid ectropion if severe enough. They can lead to restriction of neck movement and permanent mouth opening [2–4]. If left untreated in a growing child, such severe contractures can even lead to secondary facial skeletal abnormalities, compounding the problem [5]. In addition to the functional deficits facial scar can inflict, there is also the obvious social opprobrium of visible disfigurement. Scar, then, represents a significant source of morbidity, and can frequently require aggressive measures to deal with its sequelae [6].

In contrast to adults, fetal integumentary wounds in humans and other mammals heal rapidly without associated scarring until late in gestation [7–9]. Investigation into the phenomenon of fetal wound healing started in the early 1950s with the study of animal models, which showed that fetal skin wounds could heal rapidly but without any apparent “dedifferentiation” of cellular components such as occurred in regenerating amphibians [10]. Later, examination by Rowlatt [11] of healing limbs after intrauterine amputation by amniotic constriction bands in a 20-week old human fetus showed that human skin at this stage of development healed without apparent inflammation. Subsequent work has confirmed that fetal wounds heal differently depending on the gestational age of the fetus, including even in the pouch young of a marsupial [12]. In general, the scarless character of fetal wound repair persists until roughly the middle of the third trimester of intrauterine gestation, at which point a transition to the adult, scar-forming pattern of wound repair occurs [13–15]. This scarless healing is a property intrinsic to fetal tissues, and not a conferred benefit of the protected uterine environment: fetal skin placed subcutaneously into athymic mice and then wounded still heals without scar, in spite of occurring in an environment free of amniotic fluid [16]. Conversely, adult skin grafted onto immunoprivileged fetal hosts in utero and then wounded still heals with scar [17].

Because early- to mid-gestational fetal wound healing occurs with evident restoration of normal skin architecture and no significant scar deposition, it has been termed “regenerative,” and has been taken as a model by which we may attempt to engineer the same process in adults. It therefore becomes important to understand at the cellular and molecular level the distinctions between these two physiologies, in the hopes that an understanding of fetal biology may one day enable its recapitulation in the adult.

## 2. The Biology of Adult Wound Healing

### 2.1. Inflammatory Phase

The process of skin wound repair in adult mammalian organisms is an intricate and highly coordinated process that generally can be divided into four overlapping phases: hemostasis, inflammation, proliferation, and remodeling [18]. Any injury that severs blood vessels will trigger events that try to effect immediate hemostasis. This process includes vasoconstriction, platelet aggregation, and platelet *α*-degranulation of vesicles containing both clotting and growth factors. Platelets are also believed to play an additional role in the wound healing cascade, not only as initiators of coagulation but also through the release of a multitude of growth factors and cytokines that modulate fibroblast activity, such as transforming growth factor *β*1 (TGF-*β*1) and platelet-derived growth factor (PDGF) [19]. These growth factors provide a chemotactic stimulus for neutrophils, fibroblasts, and monocytes and ultimately affect the dynamics of extracellular matrix (ECM) synthesis [20, 21]. Neutrophils are early responders to these chemotactic agents and begin to infiltrate to the site of injury well before the activation and/or arrival of fibroblasts and monocytes. They accomplish phagocytosis of bacteria and functional debridement of injured tissue while themselves secreting additional proinflammatory cytokines. In the presence of foreign body or infection, a persistent neutrophil-rich inflammatory response results which can lead to poor wound healing and excess fibrosis [22]. 

Monocytes are also attracted to the wound site in response to a variety of chemoattractants derived from intra- and extravascular sources. Monocytes become macrophages, which are considered the principle coordinators of adult wound healing [23]. Macrophages act as avid phagocytes, ingesting debris in the wound field (including even spent neutrophils), and they also produce numerous cytokines and growth factors crucial for fibroblast recruitment and angiogenesis. Monocytes and activated macrophages are known to bind to the ECM through cell surface integrin receptors; this adherence to the ECM induces ECM phagocytosis, promoting wound debridement. Attachment to the ECM also alters the gene expression profile of macrophages, leading to increased expression and subsequent secretion of colony stimulating factor 1 (CSF-1, required for macrophage survival), tumor necrosis factor (TNF-*α*, inflammatory cytokine), and PDGF (chemotactic agent for fibroblasts) [24, 25]. As in the case of an extended neutrophilic infiltrate, a persistent macrophage response may also lead to excess scar formation, itself an unwanted outcome [26]. 

Another key leukocyte lineage, the mast cell (MC), derived from circulating basophils, is postulated to contribute to the healing of skin wounds, and MC's have been implicated in multiple phases of wound healing [27, 28]. Egozi et al. [29] showed using MC-deficient *Kit^W^*/*Kit^W-v^* mice that neutrophil infiltration was reduced at early time points (inflammatory phase) but that the absence of mast cells had no effect on the proliferative aspects of wound healing. Weller et al. [30] showed using the same MC-deficient *Kit^W^*/*Kit^W-v^* mice that MC activation and histamine release are required for proper recruitment of neutrophils and also for the normal closure of wounds. Ultimately, fibroblasts enter the wound site and replace the initial wound fibrin matrix by depositing glycosaminoglycans, proteoglycans, and other ECM proteins such as fibronectin and tenascin. This last is another example of the complicated interplay of multiple factors in cutaneous wound healing and scar formation, since fibronectin promotes cellular adhesion to the underlying substratum, whereas tenascin actually facilitates fibroblast migration by antagonizing fibronectin [31, 32]. 

Interestingly, although inflammatory cells are intimately involved in the regulation and progression of normal adult wound healing, several lines of evidence suggest that depletion of one or more of the inflammatory cell types can actually have a positive outcome on the closure of wounds. Experiments conducted by Szpaderska et al. [33] demonstrate that, as long as bleeding is adequately controlled, mice dosed with antiplatelet antisera to induce thrombocytopenia show no deficit in the proliferative aspects of repair, including wound closure, angiogenesis, and collagen synthesis, all of which were unaffected compared to controls. This data suggests that platelets are not absolutely essential for normal adult wound healing to occur. Similar experiments in neutrophil-depleted mice [34] showed that wound closure was actually more rapid in the mice with induced neutropenia than in control animals, suggesting that neutrophils, while perhaps highly utile in combating infection, may in other respects actually be inhibitory to wound healing. Neonatal PU.1-knockout mice (which lack macrophages and functioning neutrophils) healed wounds with minimal scarring, with an altered growth factor and cytokine profile at the wound site, reduced cell death, and with phagocytic fibroblasts standing in for more conventional inflammatory cells [35]. It therefore appears that macrophages too are not absolutely required for normal adult wound repair, and their absence may actually allow a more scarless mode of healing.

### 2.2. Proliferative Phase

The formation of granulation tissue, a well-vascularized connective tissue containing macrophages and fibroblasts that replaces fibrin clot, is a notable feature of the proliferative phase of adult wound healing. This granulation tissue has been considered to be a contractile organ, responsible for the active wound contraction seen in the proliferative phase of adult wound healing, a contraction affected chiefly by fibroblasts and their derivative subtypes, myofibroblasts (see below). The rate of granulation tissue formation appears to be dependent on interaction of the fibroblast integrin receptor with fibronectin [36]. The initial fibrin clot functions as a chemokine to stimulate macrophages and fibroblasts to migrate into the wound space; in the case of the latter, this migration itself is thought to apply traction to the wound periphery, assisting in its contraction and ultimate closure. Within the wound bed macrophages provide a continuing source of growth factors necessary for angiogenesis [37], and fibroblasts lay down a provisional matrix mainly composed of collagen and proteoglycans. 

Multiple studies have pointed to a particularly important role for transforming growth factor- beta in this phase of wound healing. Expression of the TGF-*β*1 and TGF-*β*2 isoforms (in comparison to TGF-*β*3) is increased in adult wounds, and studies show that exogenous administration of TGF-*β*1 and TGF-*β*2 to healing wounds results in increased collagen, protein, and inflammatory cell accumulation [38]. In contrast, Ferguson et al. (2009) [39] assessed scar quality after treatment with avotermin (recombinant human TGF-*β*_3_) in Phase II clinical trials in humans, and showed that avotermin has the potential to provide an improvement in the appearance of scars. As repair progresses, fibroblasts also display increased expression levels of adhesion molecules and assume a contractile myofibroblast phenotype with increased alpha-smooth muscle actin (*α*-SMA) expression, known to be stimulated by TGF-*β*1 and TGF-*β*2 as well as by PDGF [40, 41]. Wound fibroblasts and myofibroblasts work in concert to draw the wound closed and also contribute to the synthesis and alignment of collagen fibers [42].

The formation of granulation tissue in an open wound also allows the process of re-epithelialization to begin, as epithelial cells migrate across the new tissue to form a barrier between the wound and the environment. Intracellular actin microfilaments are formed, and alterations in intermediate filament gene and protein regulation have also been observed, [43] enabling the epidermal cells to creep across the wound surface. Multiple growth factors, including epidermal growth factor (EGF), keratinocyte growth factor (KGF), transforming growth factor-alpha (TGF-*α*), and epiregulin are thought to act synergistically to stimulate re-epithelialization [44–46].

### 2.3. Remodeling Phase

In the remodeling phase, which can last for up to a year after injury, there is ongoing synthesis, degradation, cross-linking, and reorientation of collagen to form the mature scar. The healing and remodeling tissue will manifest increasing tensile strength, however, the resultant scar will never attain physical properties equal to that of the uninjured tissue [47]. Remodeling allows for some removal of accumulated connective tissue and is made possible by matrix metalloproteinases (MMPs) under the control of a cytokine network [48]. The coordinated regulation of these enzymes and their inhibitors ensures tight control of local proteolytic activity. 

Over time the quantity of fibroblasts and myofibroblasts within the maturing wound bed are reduced by apoptosis, which may be precipitated by the withdrawal of cytokines as the wound heals, although the precise mechanisms governing this response remain unclear [49]. With continued remodeling the outgrowth of capillaries is halted, blood flow to the area is reduced and metabolic activity in the area declines with the maturation of a relatively hypocellular and hypovascular scar.

## 3. Disorders of Excessive Cutaneous Fibrosis

While the above summarizes many of the important features of normal adult wound healing, there also exist conditions marked by an abnormal pathological response to cutaneous wound healing resulting in excessive fibrosis, the two main examples of which are hypertrophic scars and keloids. Hypertrophic scars typically take the form of a reddish raised lump on the skin; they remain within the boundaries of the original zone of injury and often naturally improve in appearance after some years [50, 51]. They are frequently amenable to surgical revision, with the expectation that the revision will obtain a more favorable result. In contrast keloidal scars will typically progress into large, tumorous (although benign) masses that clearly exceed the original zone of injury. Keloids are not known to regress spontaneously and their recurrence rate is high after surgical excision [51], even with the use of such adjunctive measures as local corticosteroid injection or pressure appliances. Both hypertrophic scars and keloids represent a type of abnormal fibroproliferative wound repair, and it appears that several stages of wound healing, from the inflammatory phase to the remodeling phase, are significantly altered [52, 53]. 

Gene profiling studies have identified previously unsuspected genes of potential relevance to the pathogenesis of hypertrophic scars and keloids. cDNA microarray examination of tissue mRNA obtained from burn hypertrophic scars (compared to normal skin) revealed that genes displaying altered expression included proto-oncogenes, genes involved with apoptosis, immune regulatory genes, cytoskeletal elements, and transcription factors, signifying that multiple pathways are involved in hypertrophic scar formation and contraction [54]. Similar microarray studies examining altered gene expression in keloids and in fibroblasts derived from keloid lesions identified genes which have previously been noted by biochemical studies, but interestingly also identified multiple genes not previously suspected to play a role in keloid formation. Naitoh et al. [55] observed stronger ectopic expression (in keloids) of chondrocyte/osteoblast marker genes, namely, periostin, OB-cadherin, lumican, and mimecan as well as increased expression of transcription factors SOX9 and CBFA1, known to be involved in regulation of the above-mentioned gene products. The same study also marked upregulation of two tumorigenesis- related genes, p311 and fibroblast activation protein alpha (FAP-*α*). Satish et al. [56] also showed the upregulation of tumor-related genes in keloid fibroblasts, namely, TCTP (tumor protein translationally controlled 1), MORF-related gene 15 (MRF 15), annexin 2, and ribosomal proteins RPS 18, 10, and L23A. This interesting increase in genes related to tumorigenesis is congruent with the behavior of keloidal fibroblasts, which proliferate in a rapid and unregulated manner and invade normal skin tissues, beyond the boundaries of the initial region of injury.

## 4. Characteristics of Fetal Skin

In order to understand the basis of regenerative fetal skin healing, and to contrast it to the adult tissue repair described above, it is helpful to first recognize the pattern of development of fetal skin. In humans, prior to the 24th week of gestation, fetal skin tissue is less differentiated than adult skin [15]. The human fetal integument begins with two cell layers, the basal cell layer and the periderm, at about 4 weeks gestation. The periderm is the outermost single-cell layer of the fetal skin; although the function of the periderm has not been determined, a secretory or absorptive process has been hypothesized [14]. As development continues, an intermediate epidermal cell layer develops. Keratinization begins at 9 to 10 weeks gestation, and during this period hair follicles and sebaceous glands become apparent. By 24 weeks gestation, that is, toward the end of the second trimester, at which time fetal skin wounds continue to demonstrate scarless healing, the epidermis has completely keratinized and stratified into adult morphologic layers [57]. 

Fetal skin contains fibroblasts and fetal extracellular matrix (ECM) that are distinct from adult fibroblasts and adult ECM. Fetal dermis thickens by increasing collagen content and replacing nonsulfated glycosaminoglycans with sulfated glycosaminoglycans. Fetal ECM contains higher proportions of type III collagen, chondroitin sulfate, proteoglycan, and hyaluronic acid than does adult ECM [15]. Coolen et al. [58] have recently examined multiple dermal and epidermal components of human fetal and adult skin, finding that most differences between these tissue types reside at the level of dermal ECM molecular expression. For example, elastin was present in adult dermis, but was not detected in fetal dermis. Conversely, chondroitin sulfate and fibronectin both were expressed at substantially higher levels in fetal dermis compared to adult. The expression patterns of basement membrane proteins, keratin isoforms (e.g., K10, K14, K16) and epidermal Ki-67 were not significantly different between fetal skin and adult skin biopsies. 

MMPs and tissue inhibitors of the proteolytic activity of MMPs (TIMPs), molecules that regulate ECM turnover, are also shown to be differentially regulated as fetal skin develops. Dang et al. [59] found that baseline expression of MMPs 1, 2, and 14 all increased with the transition to a scarring phenotype in fetal rat skin, with MMP2 message levels increasing by 50-fold. Nonetheless, they determined that E16 (scarlessly healing) wounds had a higher MMP to TIMP expression ratio than E19 scarring wounds. Other ECM molecules differentially expressed in developing fetal skin and wounds include decorin, a proteoglycan implicated in regulation of TGF-*β* bioactivity, which has been shown to increase during the ontogenic transition from scarless fetal healing to adult wound healing, but which is actually decreased by wounding during the scarless healing period [60]. In contrast, fibromodulin, another small interstitial proteoglycan which can bind to and modulate TGF-*β* activity, has been shown to decrease with advancing gestational age, and decreases as well when adult skin is wounded, but is actually increased in scarlessly healing fetal wounds relative to control [61]. These observations give some indication of the complicated and mixed functions ECM molecules may play in determining the scarless nature of early fetal wound repair.

## 5. The Biology of Fetal Wound Healing

Healing cutaneous wounds in mammalian fetuses shows multiple important differences from adult healing; many of these are enumerated in Table 1. Perhaps the most significant characteristic feature of scarless fetal wound healing that stands in contradistinction to adult is a significantly reduced inflammatory response [62]. A markedly diminished or minimal inflammatory response in fetal wounds has been demonstrated in multiple fetal animal models [62–65]. The absence of an acute inflammatory infiltrate in fetal wounds may partly be explained by decreased platelet aggregation and degranulation in fetal tissues; fetal platelets are also thought to release lower levels of cytokines, thereby also potentially reducing the recruitment of inflammatory cells to fetal wounds [66]. Hopkinson-Woolley et al. [67], examining embryonic and fetal mice, have found that macrophages are not normally recruited to fetal wound sites before developmental stage E14.5, but that after this transition stage there was a significant recruitment of macrophages within 12 hours of injury. Furthermore, few neutrophils are present in the fetal wound, and an age-dependent defect in the ability of fetal neutrophils to phagocytose pathogenic bacteria has been demonstrated in fetal sheep, signifying that early fetal neutrophils are physiologically distinct from those present at the end of gestation or postnatal cells [68]. Thus, there are multiple facets to the much diminished inflammatory response seen in scarlessly healing fetal wounds.

### 5.1. Growth Factor Profiles During Fetal Wound Healing

The cytokine and growth factor profile of fetal healing differs significantly from adult wound healing. Many studies have focused on TGF-*β* family members as these proteins have been shown to have a major role in fibrosis [69]. In particular, the profibrotic isoform TGF-*β*1 has been noted to be reduced in early fetal wounds, and this has been confirmed in incisional and excisional wounds in murine, rat, and human skin [70–74]. Conversely, the anti-fibrotic isoform TGF-*β*3 is found in higher levels in scarlessly healing fetal wounds [75]. In addition, TGF-*β* receptors TGF-*β*RI and TGF-*β*RII are present at lower levels in fetal wounds than in adult wounds. The relative scarcity of TGF-*β*1 is of particular relevance, as multiple studies have demonstrated that the addition of TGF-*β*1 causes scar to form in fetal skin wounds that would otherwise heal scarlessly [70–72]. 

There are also differences in other growth factor groups. Although PDGFs are initially present in both adult and fetal wounds, they disappear more rapidly in the fetal wounds. Additionally, administration of exogenous PDGF in fetal rabbits induces fibrosis, consistent with the notion that the relative transience of PDGF in fetal wounds may also play a crucial role in scarless wound healing [76]. Fibroblast growth factor (FGF) family members were also found to be differentially expressed in fetal skin both with advancing gestational age and on wounding. FGF isoforms 1, 2, 5, 7, and 10 are increased in adult cutaneous wound healing, but Dang et al. found that FGF isoforms 7 and 10 actually decreased in scarlessly healing fetal wounds, while FGF 5 showed no change. There were also wound and age-dependent changes in FGF receptor isoform levels, with the authors concluding that overall there was diminished FGF expression and signaling during scarless wound healing [77]. The role of vascular endothelial growth factor (VEGF) in scarless fetal repair is not entirely clear. Some studies have found that VEGF expression is reduced in scarless fetal wounds compared to fibrotic fetal wounds, and that addition of exogenous VEGF to scarlessly healing wounds can induce fibrosis, suggesting that VEGF has additional importance to wound healing beyond simply promoting angiogenesis [78]. However, Colwell et al. [79] found that VEGF mRNA levels were higher in E16 scarless excisional rat wounds compared to fibrotic wounds at E18. 

Interleukins have also been implicated in scarless wound healing. It has been shown that the proinflammatory mediators IL-6 and IL-8 are produced in low levels in both fetal skin and in fetal-derived fibroblasts when compared to adult skin or adult-derived fibroblasts [80]. Furthermore, fetal skin deficient in IL-10, widely considered to be an anti-inflammatory agent, heals with a scar compared to fetal skin with normal levels of IL-10, which heals scarlessly [81]. Conversely, overexpression of IL-10 in adult wounds decreases the inflammatory response and creates an environment conducive for regenerative wound healing in adult organisms [82, 83]. 

 As noted above, many growth factors and cytokines appear to vary in their expression in fetal and adult wounds. While each may be important, their overall significance may be obscured by the complexity of the cytokine mileu, including other unknown or unexamined factors acting in fetal and adult wound healing.

## 6. Fibroblasts and Myofibroblasts in Fetal Wound Healing

It has been speculated for many years that fibroblasts are the primary cell type responsible for determining whether scarless or fibrotic healing will occur (see Table 2); regenerative healing, after all, ultimately depends on the ability of fetal fibroblasts to produce and arrange new collagen and other ECM components in similar quantities and ratios to unwounded skin. Studies from Lorenz et al. [84] indicated that fetal fibroblasts were able to effect scarless healing even when transplanted to an adult environment. Of particular note: no sign of fibroblast conversion into contractile myofibroblasts has been observed at the stage of fetal wound repair where scarless healing still obtains [85]. Myofibroblasts *are* found in late gestation fetal wounds, where scar formation *does* occur after transition to an adult healing pattern. Myofibroblasts are also plentiful in adult wound repair where they resemble smooth muscle cells, and their characteristic expression of *α*-SMA mediates their ability to exert a strong contractile force [86, 87]. 

One of the chief effects of TGF-*β* on fibroblastic cells is to induce the expression of *α*-SMA and prompt conversion to the myofibroblast phenotype [88].This can also occur in fetal wounds, where the addition of exogenous TGF-*β* markedly increases the abundance of myofibroblastic cells while simultaneously inducing fibrosis in an otherwise scarlessly healing fetal milieu [89]. Our own investigations have confirmed that fetal fibroblasts in culture express substantially lower levels of *α*-SMA (even with serum stimulation) than do adult fibroblasts, whereas we find no difference in total cellular *β*-actin between fetal and adult cells [90]. These observations collectively support the association of myofibroblasts with scar formation, and suggest that the lack of myofibroblasts typical of scarlessly healing fetal wounds is a critical component thereof.

## 7. Keratinocytes and Re-Epithelialization inFetal Wound Healing

Much attention has been given to the chemistry and biology of fetal dermal constituents, since that is where fetal fibroblasts reside. Relatively less attention has been paid to the properties of fetal keratinocytes and the process of fetal re-epithelialization, although it has been observed to occur more rapidly in healing fetal wounds [91–94]. Martin and his colleagues [85, 95] have focused much of their effort on fetal wound re-epithelialization and have identified fundamental differences in the mechanics of re-epithelialization in embryonic wounds. Whereas adult wounds have been shown to re-epithelialize through extension of lamellipodia followed by epidermal cells at the wound edge crawling over the wound bed [96], embryonic wounds exhibit no signs of lamellipodia or filopodial extensions. Instead, epidermal cells at the edge of wounds in both chick and mouse embryos assemble an actin “cable” that functions like a purse-string to close the wound [85, 95]. The importance of this purse-string formation was shown by the addition of cytochalasin D, which disrupted the assembly of the actin cable and blocked wound re-epithelialization [96]. Studies have also demonstrated the requirement for the small GTP-binding protein Rho, but not Rac, in the proper assembly of the actin cable and re-epithelialization of fetal wounds [97]. The presence of actin cables (and the essential role of the actin cytoskeleton) in re-epithelialization have been confirmed in fetal rat E17 wounds (at which point healing is still scarless) but not in E19 wounds (at which point the transition to adult wound healing has already occurred) [98]. In addition, paxillin has been shown to colocalize with actin in E17 wounds but not in E19 wounds, whereas gelsolin was associated with actin in E19 wounds but not E17 wounds [98]. 

Another factor that has been implicated in the faster re-epithelialization seen in fetal wounds is the more rapid upregulation of integrins in response to wounding in fetal keratinocytes versus adult keratinocytes. In human fetal skin transplanted subcutaneously onto nude mice and then wounded, increased expression of multiple integrins recognizing collagen, fibronectin, laminin, and tenascin was evident at the epidermal edge within four hours of wounding and persisted until re-epithelialization was complete [99]. In contrast, studies on adult wounds in a porcine model showed that integrin fibronectin receptors were not upregulated until 5 days after wounding [100]. Two additional studies from Zambruno et al. [101] and Juhasz et al. [102] showed that integrin expression for collagen, fibronectin and laminin receptors was not upregulated until 48 hours after wounding in healing 3-mm wounds in split-thickness adult human skin grafts transplanted onto nude mice. Thus, it appears that similar integrins are stimulated in both fetal and adult wounds but the timing at which they appear varies greatly. Reports have also identified differences in the expression patterns of the transcription factor c-fos and AP-1 in epidermis during fetal wound healing [103]. These multiple observations collectively show that the process of wound re-epithelialization and the physiology of fetal keratinocytes differ substantially from adult wound healing biology.

## 8. Expressomic Evaluation of Scarless versus Scarring Wounds

In the above sections we have reviewed many of the gene products found to display differential expression in fetal and adult wound healing, focusing mostly on growth factors and ECM constituents. However, multiple other genes have been implicated in fetal skin development and scarless wound healing. Colwell et al. [104] determined that mRNA levels of lysyl oxidase, an enzyme that cross-links collagen and elastin, were significantly greater in E19 late-gestation wounds (that heal with a scar) in comparison to E17 early-gestation scarless wounds in mouse. The homeobox genes Msx-1, Msx-2, and Mox-1 display altered expression with increasing gestational age, with Mox-1 becoming undetectable in adult skin tissues [105].

Recognizing that there remained thousands of gene products that had not been directly assayed in healing fetal wounds, investigators have begun applying more comprehensive transcriptomic techniques to the study of scarless wound healing. Several laboratories have used microarray as a tool to identify expressomic differences during scarless repair. Chen et al. [106] used a 5,705 oligonucleotide array to examine early gestational (scarless) rat skin versus late gestational (scarring) skin. They found 53 differentially expressed genes, and directly confirmed by RT-PCR and Western blot that FGF8 and follistatin had stronger expression in early gestational skin compared to late, whereas lymphoid enhancer binding factor-1 and beta-catenin showed weaker expression. Colwell et al. [107] showed that the scarlessly healing fetal skin wound transcriptome has rapid upregulation of many genes from 1 to 24 hrs, but that by 24 hrs many fetal wound-elevated gene expression levels had already begun to reverse to baseline. They suggest that numerous gene products are involved in coordinating the regenerative pattern of healing found in early fetal wounds. Recent studies by Antony et al. [108] show rapid upregulation of neurodevelopmental genes during scarless repair on after injury days 1–3. The authors speculate that these factors may promote the survival and regeneration of peripheral neurons, which may promote a scarless pattern of repair in response to injury. 

Our own laboratory has used multiple expressomic techniques to examine the transcriptome of scarlessly healing fetal wounds, including differential display [109], PCR suppression subtraction hybridization (PCR-SSH) [110], and fetal wound-specific microarrays. Each of these techniques allows an interrogation of the fetal wound expressome without any preconceptions as to which gene products may be important. Using PCR-SSH we have identified multiple candidate genes and expressed sequence tags (ESTs) to be differentially expressed in healing fetal wounds, and also fragments of genes with no clear match in the GenBank database, suggesting that the relevant gene set to scarless fetal wound healing may be larger than anticipated [110]. Using rabbit fetal wound-specific microarrays that we ourselves constructed from a cDNA library, we found that some 20% of the recovered cDNA sequence fragments demonstrated either no homology to GenBank sequences or had such limited homology that we could not confidently identify a known cognate gene (Kathju et al., manuscript in preparation). While some of these putative unknown gene products may well turn out to be species-specific variations of actual known genes, we have also obtained full length clones of several previously uncharacterized sequences, again suggesting that the determinative fetal wound gene set includes novel factors. 

Assay of the scarless wound transcriptome by differential display in our lab identified the eta subunit of the chaperonin containing T-complex polypeptide (CCT-eta) as a gene product specifically downregulated in fetal wound healing. We have subsequently shown that this pattern of expression is not shared by any other CCT subunit (of which there are eight), and that CCT-eta is less abundant in fetal fibroblasts than in adult cells [90, 111] We have also demonstrated that CCT-eta is a specific regulator of fibroblast motility and contractility, with siRNA-mediated reduction of CCT-eta inhibiting the ability of adult fibroblasts to respond to migratory and contractile stimuli, rendering them more “fetal-like” in their behavior, possibly by secondary inhibition of *α*-SMA protein expression. This observation suggests that gene products that control fibroblast motility and contractility may be especially relevant to distinguishing scarless from fibrotic wound healing [90].

## 9. Future Perspectives

Through the past 50 years, studies have been directed towards explicating the remarkable ability of mammalian fetuses to heal cutaneous wounds by regeneration, but we are still far from a complete understanding of the critical molecular determinants of this phenomenon. Although much has been learned, there remain numerous unexplored questions about the relative biology of fetal wounds versus their scirrhous adult counterparts. The role of nitric oxide, increasingly thought to be important to adult wound healing, is still largely a mystery in fetal wounds. The role of microRNAs, now also emerging as important players in adult wound healing [112] similarly remains unexplored. Ultimately, a fuller appreciation of the most important factors governing the scarless pattern of fetal wound repair will hopefully allow for intervention in the adult wound healing milieu to mitigate scar formation and improve the clinical outcome of those afflicted with the morbidity of scar.

## Figures and Tables

**Table 1:** Characteristic differences observed between fetal and adult wound healing.

	Fetal Wound Healing	Adult Wound Healing
Inflammation	Minimal	Robust

Select Growth Factors		
*PDGF*	Transient	Sustained
*FGF*	Low	High
*TGF*β**	Low TGF*β*1 and TGF*β*2	High TGF*β*1 and TGF*β*2
	High TGF*β*3	Low TGF*β*3

Proinflammatory cytokines		
*IL-6*	Low	High
*IL-8*	Low	High

Anti-inflammatory cytokine IL-10	High	Low

Formation of granulation tissue	No	Yes

ECM Proteins		
*Collagen*	Increased Type III collagen (ratio of type III : type I is high)	Increased Type I collagen (ratio of type I : type III is high)
*Fibronectin*	Early Deposition	Late Deposition
*Tenascin*	Early Appearance	Late Appearance

Proteoglycans and Glycosaminoglycans		
*Hyaluronic Acid*	High	Low
*Fibromodulin*	High	Low
*Decorin*	Low	High

Myofibroblasts	No	Yes

Integrins during re-epithelialization	Early	Late

CCT-subunits		
*CCT-eta*	Low	High
*CCT-beta*	No change	No change

**Table 2:** Observed differences between fetal and adult fibroblasts.

	Fetal Fibroblast	Adult Fibroblast
alpha-SMA (serum containing cultures)	Low	High

In-vitro collagen contraction	Less	More

Hyaluronic acid (HA)	Increased HA synthesis irrespective of cell density	Decreased HA synthesis as cell density increases
Hyaluronic acid receptor	High	Low

Proinflammatory cytokines		
*IL-6*	Low	High
*IL-8*	Low	High

CCT-subunits		
*CCT-eta*	Low	High
*CCT-beta*	No change	No change
